# Perspectives on the insidious nature of pain metaphor: we literally need to change our metaphors

**DOI:** 10.3389/fpain.2023.1224139

**Published:** 2023-09-15

**Authors:** Mark I. Johnson, Matt Hudson, Cormac G. Ryan

**Affiliations:** ^1^ Centre for Pain Research, School of Health, Leeds Beckett University, Leeds, United Kingdom; ^2^ Mind Help Limited, Durham, United Kingdom; ^3^ Centre for Rehabilitation, Teesside University, Middlesbrough, United Kingdom; ^4^ The Pain Education Team Aspiring Learning (PETAL) Collaboration

**Keywords:** pain, metaphor, linguistic relativity, pain language, enactive metaphor, lived experience, salutogenesis, painogenicity

## Abstract

Metaphorical language is used to convey one thing as representative or symbolic of something else. Metaphor is used in figurative language but is much more than a means of delivering “poetic imagination”. A metaphor is a conceptual tool for categorising, organizing, thinking about, and ultimately shaping reality. Thus, metaphor underpins the way humans think. Our viewpoint is that metaphorical thought and communication contribute to “painogenicity”, the tendency of socio-ecological environments (settings) to promote the persistence of pain. In this perspectives article, we explore the insidious nature of metaphor used in pain language and conceptual models of pain. We explain how metaphor shapes mental organisation to govern the way humans perceive, navigate and gain insight into the nature of the world, i.e., creating experience. We explain how people use metaphors to “project” their private sensations, feelings, and thoughts onto objects and events in the external world. This helps people to understand their pain and promotes sharing of pain experience with others, including health care professionals. We explore the insidious nature of “warmongering” and damage-based metaphors in daily parlance and demonstrate how this is detrimental to health and wellbeing. We explore how metaphors shape the development and communication of complex, abstract ideas, theories, and models and how scientific understanding of pain is metaphorical in nature. We argue that overly simplistic neuro-mechanistic metaphors of pain contribute to fallacies and misnomers and an unhealthy focus on biomedical research, in the hope of developing medical interventions that “prevent pain transmission [sic]”. We advocate reconfiguring pain language towards constructive metaphors that foster a salutogenic view of pain, focusing on health and well-being. We advocate reconfiguring metaphors to align with contemporary pain science, to encourage acceptance of non-medicalised strategies to aid health and well-being. We explore the role of enactive metaphors to facilitate reconfiguration. We conclude that being cognisant of the pervasive nature of metaphors will assist progress toward a more coherent conceptual understanding of pain and the use of healthier pain language. We hope our article catalyses debate and reflection.

## Introduction

“Disciplines progress according to the strength of their metaphors, and those metaphors are fated to become so familiar that they transform into illusions, if even thought of at all.” (1) p.3

Most people understand metaphors according to classical theories of language, as expressions used for figurative embellishment of objective and literal modes of representation. In 1980, Lakoff and Johnson argued that metaphors were more than a literary characteristic of language delivering “poetic imagination and rhetoric flourish”. They claimed metaphors underpinned the way humans *think*; thus, common concepts encoding knowledge were built using metaphoric structure (2). Nowadays, metaphor is considered a basic tool of cognition to comprehend abstract concepts and perform abstract reasoning. In other words, metaphor is fundamental to the way humans frame sociocultural knowledge and structure conceptual systems (3). The Sapir–Whorf hypothesis of linguistic relativity (linguistic determinism) proposes that the structure of a person's native language and culture shapes how they construct their living experience (3, 4, 5, 6, 7, 8). Thus, a person's language and cultural narrative are likely to influence whether a person's living experience of pain is associated with health, illness, suffering, and whether there is potential for recovery and/or living well with pain.

Our viewpoint is that metaphorical language and metaphorical thought contributes to “painogenicity”, the tendency of socio-ecological environments (settings) to promote the persistence of pain (9). Painogenicity reflects the sum of influences that the surroundings, opportunities or conditions of life have on the persistence, severity, and impact of pain, on individuals, groups, and communities (9, 10). Painogenicity, acknowledges micro, meso and macro level influences, especially modern-day social, environmental, and commercial conditions, that make pain “sticky” (11). Previously, we have argued that a salutogenic healthy settings approach may ameliorate painogenicity and reduce the burden of persistent pain on society (12). Salutogenesis (Latin “salus” meaning health; Greek “genesis” meaning origin) is defined as the study of factors that support health as opposed to factors causing disease (13). Salutogenesis explores how people cope with stressors in daily life to remain physically and emotionally healthy (14). Central to salutogenesis is the concept of “sense of coherence”, a dispositional orientation allowing a person to be resilient to life-stressors to maintain and improve health and well-being, consisting of comprehension, manageability and meaningfulness of their experiences (e.g., pain) (15, 16). Pivotal to salutogenesis are generalized resistance resources to cope effectively with situations (e.g., money, knowledge, coping strategies, social network). Thus, salutogenic approaches focus on building systems that support a person's sense of purpose and meaning in their life. Salutogenesis is underpinned by “whole person health” (17) arising from the “Whole Person Medicine” movement in the 1970's (18). Oliveira advocates a salutogenic approach to the education of individuals and society about pain, including positive aspects of suffering to improve a person's sense of coherence (19). Thus, constructive and positive pain narrative is the foundation of salutogenesis and whole person health.

The aim of this article is to explore the insidious nature of metaphor in pain language and conceptual (biomedical) models of pain and its persistence. We use the lens of linguistic relativity to reveal the insidious nature of pain language and argue a need to reconfigure metaphor to align with contemporary models of pain experience and salutogenic approaches to living well with and without pain. It is not our intention to undertake a comprehensive review but to challenge dogma and raise issues for scholarly debate. The foundation of our article is based on a free text search of PubMed (“[pain (all fields)] AND [metaphor (all fields)]”, 04 October 2022, 207 items) and additional literature found therefrom. Our review is narrative and based on literature that we believed was relevant, contradictory, and contentious. We acknowledge that this approach is open to selection and evaluation biases and opinion-based arguments. Readers are encouraged to follow up references for comprehensive coverage of issues. Before discussing the insidious nature of pain metaphors, it is important to contextualise how metaphors shape human reality.

## How metaphors shape human reality

### What are metaphors?

People use various types of figurative language to express ideas, abstract concepts, inner experiences, and comparisons.

Aristotle defined metaphor as: “*giving something a name that belongs to something else; the transference (“epi-phora”) being either from genus to species, or from species to genus, or from species to species, or on the grounds of analogy … metaphors are constituted on the basis of our ability to see the similarity in dissimilars”* (20) p.4 [citing Aristotle (21)].

The Oxford Dictionary defines a metaphor as “*a word or phrase used to describe somebody/something else, in a way that is different from its normal use, in order to show that the two things have the same qualities and to make the description more powerful, for example She has a heart of stone”* (*22*).

Thus, metaphors apply a word or phrase to an object or action to which it is not factually appropriate, to convey one thing as representative or symbolic of something else. For example, “pain is a knife stabbing my leg”—pain is *not actually* a knife; or “there is a gnawing pain in my bone”—pain is not actually gnawing the bone. In metaphor, the properties of one thing are integrated with the other, leaving the observer to interpret the relationship.

Linguistically, metaphors are distinct from other types of figurative language such as:

• Simile—comparing one thing with another thing using the words “like”, “as”, “so”, or “than”. Simile makes explicit the fact that the properties of one thing are alike to the properties of another thing (i.e., the two distinct entities are not the same) e.g. “my pain was like a rat, gnawing at my bone” or “my hand feels like it is a burning glove”.
• Metonymy—a word or name used to refer to a thing closely associated with another thing, e.g. referring to the quality of pain as “the gnawing continued”
• Hyperbole—exaggerated statements or claims not meant to be taken literally, e.g. “the pain is a million times worse than other pains I have had”
• Idioms—a group of words that has a different meaning than each word on its own, e.g. “a pain in the neck”—which refers to an irritating person, thing, or activity, rather than a neck that is actually painful.

In terms of utility, linguistic precision is of limited importance in conceptualisation and communication of pain, providing concepts remain correct. For the purposes of this article, we will use the word “metaphor” to encompass all types of figurative language that makes an implicit comparison of two things that are similar but not the same. Precise linguistic terminology will only be used when it affects the specific meaning of arguments, e.g., “Pain is like electric shocks shooting down my leg” will be described loosely as “metaphor” (metaphorical language) rather than its precise linguistic definition as a simile.

### Metaphorical thinking

In 1980, Lakoff and Johnson reasoned that metaphors used in everyday conversation enable understanding and expression of abstract concepts, such as feelings or ideas, by the process of making sense of one type of thing in terms of another (2). Lackoff and Johnson stated:

“Our concepts structure what we perceive, how we get around the world, and how we relate to other people. Our conceptual system thus plays a central role in defining our everyday realities. If we are right in suggesting that our conceptual system is largely metaphorical, then the way we think, what we experience, and what we do every day, is very much a matter of metaphor. But our conceptual system is not something we are normally aware of. In most of the things we do every day, we simply think and act more or less automatically along certain lines. Just what these lines are is by no means obvious. One way to find out is by looking at language. Since communication is based on the same conceptual system that we use in thinking and acting, language is an important source of evidence for what that system is like.” (2) p.3.

Thus, metaphors are a conceptual tool for categorising, organizing, thinking about, and ultimately shaping reality; this is known as the cognitive metaphor theory (3). Since 1980, scholarship on conceptual metaphor theory has developed within the larger disciplines of cognitive linguistics and cognitive psychology. Claims of conceptual ambiguities and challenges to the accuracy of empirical evidence means that cognitive metaphor theory has evolved over time, although the central concept remains irrefutable (23).

Importantly, Lakoff and Johnson demonstrated that metaphorical concepts are formed according to the configuration of a human body interacting with the external environment, i.e., as a 3-dimensional object in space acting consciously within the dimension of time. Consequently, conceptual thinking has developed according to the constraints of being human, i.e., a visually dominant, bipedal, upright, mobile human being living on the surface of a spherical planet under the force of gravity. Human concepts develop according to the human body schema and are characterised by front, back, top, bottom, middle (medial), side (lateral), left, right, inside, and outside, and in relation to moving forward, backwards, up, and down. It is unlikely that these concepts would develop in a sentient organism with a spherical body-schema existing in the gravity-free void of space (24). Consequently, the constraints of embodied human existence restricts and obscures human conceptual thinking.

### Metaphor shapes who we are

Perceiving, acting and communicating in metaphorical language shapes mental organization and is realised through embodied neural circuitry that encodes signatures of conceptual domains, as described in the neural theory of metaphors [for review see (25)]. Metaphors are at the core of our lived experience, they govern the way we perceive, navigate and gain insight to the nature of the world, creating and describing new realities (26). Metaphor is a tool to project private experiences (sensations, feelings, and thoughts) onto externally located objects and events to understand one's own inner bodily state and to communicate this private inner experience to others.

The process of creating coherently organised experience through metaphor involves the use of a source domain that is shared by others (e.g., an enemy) to understand a target (concept) domain (e.g., pain). Thus, the idea that “pain is an enemy” comprises a concept we are trying to understand (e.g., *pain*) and a concept from which we draw a metaphorical expression (e.g., *an enemy*). “Pain is an enemy” is considered a *primary* metaphor because it forms a “rudimentary theme” that spawns secondary metaphors such as “fighting pain”, “battling pain”, “surrendering to pain”, and “pain killers”.

## The utility of pain metaphor

### Metaphor to understand pain in oneself

Pain is a complex, sometimes formless, bodily experience not directly sharable to others. Humans describe formless sensations and feelings by “projecting” the experience to objects and events that have form in the external world. Thus, people borrow from the world of form and meaning to connect bodily symptoms to objects, enabling symptoms to gain a sense of structure, i.e., they apply a word or phrase to convey one thing as representative or symbolic of something else.

Using language to “project” inner states to entities and events in the external world helps people gain a sense of clarity and control of the meaning of their experience. A thematic analysis of interviews with 23 older adults by Clarke et al. (27) revealed that people use vivid stories, metaphors, and similes, rather than using isolated words, to personalise the meaning of their lived experience of chronic pain, e.g., “two bones rubbing together” or ‘the sensation of “running cold water”. Nortvedt and Engelsrud (28) found men used dramatic metaphors to describe the impact of phantom pain sensation on relationships with their self (body), others, and the world, e.g., “being invaded by insects” or “skin being scorched and stripped from the body”.

In the book *The Language of Pain*, Biro describes metaphors as a powerful means of worldmaking, creating a descriptive language for the often silencing effect of pain (29).

“In pain, we don't choose metaphor but are forced in that direction because there is no literal language; it's either metaphor or continued absence of speech.” (29) p.73

Biro argues that metaphor is the only means available to represent the reality of pain experience.

“Pain threatens to destroy our language and conceptual abilities, leaving a void. The only way to represent the experience and fill the void is through metaphor.” (29) p.75

In this quote, Biro transforms pain into a “thing” that threatens to destroy. Thus, metaphors attempt to objectify the subjective. Bourke describes this as a metaphorical *concretisation of pain* that brings the nature of private experience into the “knowable, external world” of others.

“Metaphors enable people to move a subject (in this case, pain) from inchoateness [not yet properly developed] to concreteness” (30) p.477.

The idea of pain as a “*concrete thing*” is contentious and exposes ongoing tension about the nature of pain and the use of literal and metaphorical language (31, 32, 33). Bourke contends that pain should be considered as a “kind of event” or “a way of being-in-the-world” (30, 34). To make sense of “unstable pain-events” people constitute and reconstitute their experiences of the body's behaviour during and after social and environmental interactions using metaphorical language. Thus,

“… bodies are not simply receptacles of sensations, but are actively engaged in the linguistic processes and social interactions that constitute those sensations” (30). p.475.

### Metaphors to share pain with others

Explaining the experience of pain is likened to making the invisible visible. Metaphorical expression uses a common understanding of words and non-verbal vocabularies e.g., visual art, music, and rhythmic movement, to communicate pain, and to elicit an empathetic response. Semino (35) summarised psycholinguistic and neuroscientific research that supports the premise that detail, creativity and textual complexity of pain metaphor can influence the nature and intensity of an embodied simulation of pain experience, a proxy of empathy, in listeners.

Metaphoric communication of pain enables the expression of disordered and indescribable inner thoughts and feelings providing emotional release and relief. Shinebourne and Smith (36) suggest that metaphors provide a “safe bridge” to communicate emotions too distressing to express literally. Metaphoric expression enables the repair of broken connections of the internal sense of self and with oneself, culture, and society. Sharing experiences creates a sense of “connective liberation”. McFarland et al. (37), argue that living with pain is an “emotional time bomb” and that metaphoric thinking can help to deactivate and reframe inner emotions, and “off-load” the explosive and destructive inner experience of living with pain to oneself and to others.

### Metaphor during clinical consultation

In health care settings, scaffolding for a person's sense-making of their bodily experience comes from a variety of sources such as the physical and social environment of the clinic, and the consultation with practitioners. During a consultation, patients describe their internal states using stories flooded with metaphors. Thus, metaphorical dialogue between patient and practitioner is the norm, although neither is fully aware that they are talking in metaphor (just as we have done here by instinctively using the term “flooded”).

A thematic analysis of 18 interviews of pain practitioners by Munday et al. (38) revealed that metaphors were used as a communicative tool, a clue, an obstacle and as an adjunct in treatment. Practitioners reported most metaphors used by patients provided insight into the meaning attributed to pain, although some metaphors used by patients were unhelpful for patient recovery. Sometimes interpreting metaphorical meaning in patient narrative was challenging because patients were using metaphors that did not align with the clinician's biomedical paradigm.

Often, healthcare professionals default to structural biomechanistic metaphors when explaining pain, perhaps because it is an easier viewpoint from which to understand the body. However, biomechanistic metaphors may infer, whether intentionally or not, that the body is damaged, fragile, weak, and slow to heal. Metaphorical language can conjure up distressing imagery such as “bone-on-bone” or “wear and tear”. People may interpret metaphors literally, believing that vertebral discs “slip”, core stability has “gone”, or joints have “seized”. These metaphorical misunderstandings and incorrect beliefs may reinforce rumination on sensations of pain and stiffness, attention to crepitus, and fear avoidance of movement. Medical imaging used to confirm pathology and anatomical models used to explain pathophysiological processes may inadvertently strengthen negative rumination. The use of a destructive pain metaphor is insidious and generally goes unnoticed.

## Insidious metaphor to describe pain?

Destructive metaphor aligns with explanatory models associating pain with actual or potential tissue damage. This reinforces the allure of biomedical, pathoanatomically based remedies. This may impede engagement with first-line health promoting (salutogenic) self-managed lifestyle adjustments of physical activity, diet, and positive psychological state. The use of destructive pain language conjures up metaphors of warfare that dates to antiquity, and are so ingrained that it may be very difficult to change.

### Warmongering metaphor for pain

Historical analyses of pain metaphors provide useful insights into how the societal meaning of pain has changed through the ages (30). A prevailing view throughout early history, as described by the Greek physician Galen AD 129–216, was that pain was “of the soul”, associated with illness and disease resulting from an imbalance of internal humors.

Díaz Vera (39) appraised metaphorical language in Middle English medical writings from the period 1350–1500 and found that pain was described as a process of commencement, treatment, and cure, rather than a permanent state, i.e., as a type of event [*c.f* (30, 34).]. Often pain was described (metaphorically) as a gas entering the body (“being in motion”) to affect bodily organs, or as living entities with hostile intentions e.g., a living creature that grows within the body or as an angry person outside the body. Thus, medieval physicians described “fighting pain” with an arsenal of weapons (treatments); this contrasted with religious treatises and homilies that pain needed to be endured for relief in the afterlife (40).

By the 17th century, warmongering language underpinned beliefs that relief of pain depended on correct medical treatment. The prevailing view was diseases were caused by discrete pathological entities (objects) that were “the enemy” and could be “targeted” (treated) by interventions that “eradicated”, “annihilated”, “attacked”, “battled”, and “destroyed”. The development of Germ Theory in the late 1800s, engrained warmongering metaphors within the medical discourse and people with disease began to be viewed as “clinical research material” within a “metaphorical medical battlefield”. Bourke's historical analysis of metaphorical language within medical texts of the eighteenth and nineteenth centuries reveals the attitudes of many physicians to wage war on diseased tissue with little compassion towards the person in pain (41). Thus, medical practice involved decontextualising a person's lived experience of pain.

By the 20th century, warmongering language was ubiquitous in medical literature, with healthcare practitioners (the soldiers) encouraged to “wage war” on communicable and non-communicable diseases (the enemy) such as AIDS, Covid, cancer, diabetes, obesity, and pain. The success of “an arsenal of weapons” such as antibiotics, vaccination, medication, and surgery has conceptualized the body in metaphors of warfare that are so pervasive they go unnoticed, e.g., “a battle against disease”, “winning or losing the fight”, “pathogens invading or attacking”, “the body”s defences”, “doctor”s orders”, “the magic bullet” and “fighting disease”.

### Damage metaphor for pain

Warmongering metaphors easily unite with the metaphor “pain is damage” in common language about; sensations (e.g., “attacks of pain”, “stabbing pain”), emotions (e.g., “the horror of pain”), thoughts (e.g., “tortured by pain”), treatments (e.g., “pain killers”), strategies for relief (e.g., “fighting pain”) and personnel (e.g., “victims of pain”). Pain assessment tools to capture the quality of pain are dominated by metaphors of damage and warmongering. A case in point is the McGill Pain Questionnaire (MPQ), designed to “measure” sensory, affective, and cognitive dimensions of pain. Patients are invited to describe how their pain “feels” by selecting from a list of words, examples of which include “throbbing”, “stabbing”, “shooting”, “gnawing”, “lancinating”, “burning”, “scalding”, “searing”, “stinging”, “suffocating”, “killing”, “blinding”, “penetrating”, “piercing” “tearing” and “torturing”. An online survey of 247 people with various persistent pain conditions by Munday et al. (42), found pain metaphors to be characterised by the overarching theme of “damage”, with source domains including electricity, insects, rigidity, causes of damage, bodily misperception, and death and mortality. Damage dominates the lexicon of pain.

Leading pain organisations define pain within a framework of tissue damage (e.g., the International Association for the Study of Pain (IASP), the European Federation of International Chapters of IASP (EFIC), American Pain Association, and the British Pain Association). The IASP's definition of pain is “An unpleasant sensory and emotional experience associated with, or resembling that associated with, actual or potential tissue damage” (43). Pain organisations emphasise the complex relationship between tissue damage and pain experience, i.e., it is not one-to-one; rather pain is a multifaceted sensory, emotional and cognitive experience influenced by ecological, sociological, psychological and biological factors. Thus, serious tissue damage may occur without pain (44), and pain may occur without tissue damage (45, 46, 47), although this is often counterintuitive to patients.

Warmongering metaphor is often used for motivational messaging, e.g., the British Pain Society's 2022 Pain Awareness Month campaign “*I beat cancer. Now I am fighting pain*”. There is, however, an insidious side to damage and warmongering metaphors. Fighting pain may encourage unrealistic expectations that recovery is associated with how hard you fight. Fighting fosters an ever-expanding arsenal of weaponry (e.g., painkillers) leading to the medicalization of issues that may be socially rather than biomedically rooted; instigating overdiagnosis and overtreatment (20, 48, 49, 50). Fighting pain could misdirect efforts; for example, by therapy shopping for “quick fix cures” at the expense of interdisciplinary biopsychosocial-based treatment and salutogenic approaches that promote living well with and without pain (51).

Corkhill (52) suggests that “fighting pain to the end” may be a feisty attitude but thoughts of fighting your own body may trigger psychophysiological stressors that may make pain worse. Warmongering language creates “battlefields” rather than “safe havens” and this is not only contrary to the goal of alleviating suffering and aiding recovery, but reveals issues such as “where are the safe havens and how do people get to them?” So, metaphors of damage and warmongering may harm health, well-being, and recovery by:

• Generalising conceptual (mis)understanding of pain as *always* being due to tissue damage resulting in constant vigilance, e.g., of an “attack” of pain
• Fostering thoughts of war, suffering, chaos and being unsafe resulting in fear, worry, anxiety hopelessness and despair, i.e., warzones are not conducive to recovery
• Placing people into a state of persistent fight, flight, freeze or flop, i.e., sympathetically mediated stress
• Encouraging simplified thinking of treatments as weapons to “quick-fix tissue” and practitioners as “soldiers to kill pain”
• Fostering ideas of an end game of winning or losing, leaving no space for play, laughter, curiosity, or healing in the moment

We contend that fighting pain involves placing the self in a civil war against itself, rather than creating peace through arbitration and a sharing of values.

Scarry (53) argues that reducing pain to a sign and symptom of illness, disease, or trauma places the person within a paradigm of disability, disorder, and diagnosis, reinforcing biomedical discourse and decontextualising a person's lived experience. At the core of biomedical discourse is a neuro-mechanistic model of pain conceptualised in metaphor that often goes unnoticed, is insidious in nature, and motivates patients to constantly search for medical solutions. Biomedical discourse was not always the norm.

### Erosion of culturally derived metaphors

In many so-called “traditional societies”, the language used to describe pain and discomfort has its roots in “biophilic metaphors”. These are figures of speech that draw from nature and natural processes to convey abstract ideas or emotions such as phrases that invoke images of the land, weather, or local flora and fauna. Such metaphors were not merely poetic expressions but reflections of deep-seated cultural beliefs and understandings of the human condition (54). Over time, the spread of Western mechanistic medicine in conjunction with the pervasive reach of global media, began to mould and, in some cases, outright replace these indigenous understandings of pain. Research by Halliburton suggests that traditional interpretations of mental illness that reference spirits with names and personalities in Kerala, India, have been replaced by psychological idioms such as “tension”, “stress” and “depression”—concepts that, while perhaps more standardised, lack cultural specificity or veracity (55). Halliburton advocates salutogenic approaches that incorporate traditional and biomedical modalities (56, 57, 58).

Our viewpoint is that the erosion of biophilic cultural beliefs and understandings of the human condition may foster a necrophilous mindset. A person with a “biophilic” lens sees growth, function, and the spirited intricacies of living beings, with a love for life, whereas a necrophilous person is “entranced” by static and mechanical aspects of life, drawn to the unchanging, lifeless, and inanimate, seeing living beings as objects, devoid of spirit or agency (59, 60). Interpreting pain through a necrophilous lens replaces the rich tapestry of cultural nuances with a cold, clinical uniformity. We contend that the biophilic spirit of humankind, which thrives on understanding, empathy, and the celebration of life in all its forms, is eroded by this mechanistic view, leading to a world where pain, and by extension, life, is understood not in its vibrant, multi-faceted entirety, but as a mere malfunctioning of physiological machinery.

In fact, biomedical pain language has encroached upon human activity unrelated to potential or actual tissue damage, i.e., unrelated to so-called “physical pain [sic]”. This is often termed “psychological pain” or “social pain” and refers to unpleasant experiences such as grief, sadness, anguish, embarrassment, shame, and hopelessness that arise from social situations such as the death of a loved one, rejection from a social group, or bullying. Shneidman (61) devised the neologism “psychache” to describe unbearable psychological anguish, soreness, hurt, “pain” and aching and theorized that unresolved psychache due to an unfulfilled psychological need caused suicide. This demonstrates the pervasive nature of biomedical pain language in the development of scientific concepts of the human condition.

## Insidious metaphorical concepts in pain science?

“There are no metaphor-free zones in science” (62) p.131.

Metaphor shapes scientific knowledge and the development and communication of complex, abstract ideas, theories, and models. Thus, explanatory models of pain are always metaphorical because they develop according to the constraints of human conceptual thinking.

Conceptual models of pain are described using neuro-mechanistic metaphor, comprising detectors (nociceptors), wiring maps (neural pathways), gates (synaptic processing), locks (membrane receptors), keys (neurotransmitters), doors (gated ion channels) and processing centres (ganglion and nuclei). A cornerstone of contemporary thinking from pain science is the so-called “Gate Control Theory” that uses a “gate” metaphor to represent how the flow of neural information (nerve impulses) is modulated (inhibited or facilitated) at synapses in the central nervous system (63, 64).

The search for biosignatures, such as neural correlates, is pivotal to a scientific explanation of how the subjective pain experience arises from the activity of the “stuff” (biological matter) of the body. Neuro-mechanistic explanations of nociception remain a dominant component in the biopsychosocial model of pain and exert a powerful influence on clinical practice (65, 66). Bendelow (67) contends that pain is a subjective, value-laden, sensory and emotional experience that relies on bodily signs and culturally-embedded language that is subject to multiple interpretations, and medicine reduces the complexity of pain to a system of nerve impulses signalling tissue damage. Corns (68) describes this as an “orthodoxy of simplicity” and argues, using the tools of analytic philosophy, that pain is so complex and idiosyncratic that scientific generalisations from mechanistic models may have limited utility [see also (69)]. In an appraisal of “Pain as a metaphor”, Neilson contends that the neuro-mechanistic model of pain is oversimplistic, unsophisticated and based on metaphorical short-hand that hinders a more encompassing understanding of pain (1).

Neilson (1) uses the “pain pathway [sic]”, included in every textbook of pain, as an example of the insidious nature of biomedical schematic diagrams, the “sine qua non of the medical pain discourse”. Neilson states:

“[The pain pathway] shows a peripheral stimulus sending a signal to central structures (a wire system), the diagram is conceptually as simple as Descartes” thread running from the skin to the brain: no more advanced than the Cartesian model of thread running from the skin to the brain” (1) p. 8.

Neilson contends that an unhealthy focus on neuro-mechanistic metaphor conflates nociception and pain, promoting misconceptions, such as pain being sensed, transmitted, and gated, that contaminate scientific literature. Examples of some common fallacies and misnomers include “pain-sensing neurons” (70), “… abdominal pain transmission …” (71), “Astrocytes contribute to pain gating in the spinal cord” (72). Fundamental conceptual errors remain unchecked in prestigious scientific journals in favour of incorrect metaphorical shorthand, for example, a Research Highlight in the journal *Nature* titled “Nerve cells that carry pain signals” (73). Cohen et al. (31) call for “epistemic discipline” in the use of language and logic in pain medicine to prevent fallacies and misnomers such as reification of pain (treating pain as if it were a physical entity—a “concrete thing”).

Neilson argues that the neuro-mechanistic model of pain drives a research agenda generating vast amounts of sophisticated biomedical data that “ *… create[s] an illusion of vast medical knowledge that, to a significant degree, is metaphor-based*” (1) p. 3., placing the power of authority to “police the door for pain remedies” to the medical sciences. Neilson states:

“Mechanisms are emphasised in medical discourse. ‘What is pain?’ is a difficult question to answer, but opiate and GABA receptors can be identified, tested in experiments, and the results published in articles rich with schematics and diagrams. In this way, the simple is represented simply, demonstrating the secret and dangerous power of visual representations that avoid images of human beings in pain. Standing on the shoulders of schematics, medicine appears powerful and knowledgeable. Yet the schematics are metaphors which perpetuate themselves to the detriment of complex truth. Schematics are visual metaphors that limit understanding because of extreme simplicity.” (1) p. 6.

Mechanistic metaphors pervade advertisements for pain treatments. Violet (74) analysed metaphors in commercials of pregabalin for fibromyalgia. A neuro-mechanistic metaphor reduced fibromyalgia to one symptom, pain, that travelled in a “wire”, thus reducing the person with fibromyalgia to a body part (disembodied), and pain to a “pulsating scientific aesthetic”. This is far from the realism of living with fibromyalgia. In addition, metaphors of “illness as a thief”, “fear of isolation” and expectations of “normality” were used to evoke guilt and provoke a desire for pregabalin to aid a return to gendered domestic life before illness [for further discussion of how metaphor can stigmatize people see (75, 76)].

Biomedical orthodoxy and obstinate adherence to materialistic reductionist frameworks of the Standard Model of Physics may have constrained a more encompassing understanding of pain by focussing on deconstructing systems, organs, tissues, cells, molecules, and even subatomic particles at the expense of the “whole person”. Conflating pain and nociception contribute to highly convergent research activity grounded in a “comfortable professional consensus”, reinforced by attractive biomedical metaphors. There is no doubt that the mechanistic model of pain provides incredible insight into structures and processes but has not explained subjective experience; nor reduced the burden of persistent pain.

We have argued that metaphors used in pain language are negative, destructive and insidious and we advocate reconfiguration of pain metaphors towards constructive, holistic, and person-centred. This requires a paradigm shift away from a simplified biomechanistic pain metaphor toward a salutogenic pain metaphor, reflecting a richer understanding of biopsychosocial processes and subjective phenomenon, and informed by non-biomedical disciplines. Diligence in appropriate use of language and logic is critical to reduce fallacies and misnomers that result in suboptimal patient care and potential harm (31). We acknowledge that such a shift is likely to be very slow. Moreover, it is critical to balance the precision and utility of language used to convey pain concepts, especially when assisting conceptual understanding for the lay person in community-orientated education (77). In the next section, we appraise strategies being used to reconfigure pain language.

## Reconfiguring metaphors used in pain language

### Constructive pain metaphor

The need to adopt constructive metaphorical language that reflects contemporary understanding of pain experience has been acknowledged, e.g., “*The malleable magic of metaphor*” by Moseley and Butler (78). Moseley and Butler advocate societal strategies to adopt positive pain metaphors within a psycho-educational model that re-contextualizes pain from the primary metaphor “pain is damage” to “pain is protection”. This spawns constructive metaphors such as “pain as a gift”, “sore but safe”, “hurt's not harm” and “pain is an alarm” (78, 79). “Pain is an alarm” and “Pain is a protector” have become dominant metaphors used in public health initiatives and by pain education providers to assist people reconceptualise pain, e.g., Live Well With Pain (www.livewellwithpain.co.uk), Pain Revolution (www.painrevolution.org), Flippin' Pain (www.flippinpain.co.uk), and Neuro Orthopaedic Institute Australasia (Noigroup, https://www.noigroup.com/).

Contemporary constructive metaphors concur with evolutionary theories that pain serves to warn of stimuli that cause potential or actual disruption to the integrity of the body, including stimuli that may hinder tissue healing achieved by making injured body parts “sensitive” (80, 81, 82). Pain commands attention and utilises cognitive resources to elicit behaviours that attempt to minimise physiological disruption to alleviate the pain. In situations where pain persists with no clear underlying condition or out of proportion to any observable injury or disease (primary chronic pain), metaphors of “alarm” and “protection” can be developed and stories created to assist understanding of socio-psycho-bio factors that influence pain and its persistence, e.g., “an oversensitive alarm” and “an overprotective brain” [see (83) for examples].

### Metaphorical stories

Storytelling is an essential characteristic of human beings (84). Stories with metaphors that include visual and verbal cues are increasingly being used to aid health communication and literacy, e.g., in acceptance and commitment therapy (ACT). The therapeutic effect of metaphor has been shown to improve when people imagine themselves as a protagonist of a metaphorical story compared with the story presented in the third person (85). Stones and Cole (86) developed a primary metaphor-based visualisation called the “bus of life”. The person (reader) is described as on their “bus of life” when, one day, the pain got on as a passenger; the bus can be driven by the pain or by the reader. The “bus of life” metaphor enables a sustained and coherent “big picture” narrative of emotional qualities and meaning and can spawn secondary metaphors, e.g., “direction of travel”. If pain drives the bus, a persistent “red *Pain* wheel”, indicating a persistent pain cycle, is in control of the direction of travel, whereas if the reader drives the bus, a persistent “green *Gain* wheel” is in control alluding to a virtuous circle (86).

Vilardaga et al. (87) offers other examples of storylines:

• The Football Player and the Robbery Victim to Pain: To describe 3 distinct features of chronic pain, i.e., personal relevance, complexity, and unpredictability of pain—to address hopelessness and lack of connectedness to others.
• Life Navigation System and The Fog of Pain: To introduce the importance of identifying and reconnecting with personal values—to address values clarification and behavioural activation and change.
• Life Rhythms: To introduce the mechanics of behaviour change and the importance of consistent rates of behaviour—to address pacing and behavioural momentum.

### Metaphorical images

Padfield (88) reported the benefits of using visual metaphors to facilitate dialogue in clinical consultations. Patients selected a photographic image, from an assortment, that best represented their pain experience, enabling a “shared narrative space” for practitioner and patient to negotiate the meaning of pain. Padfield et al. have found that metaphoric images catalyse memories of experiences to construct meaning, increase disclosure of emotional information from the patient and increase empathetic engagement from the clinician (88, 89, 90).

Stilwell et al. (91) created five paintings of pain-related metaphors from a study of sense-making of pain during communication between patient and clinician. The paintings were then used to catalyse deeper levels of reflection on the language, action, meaning, and experience of pain. This process revealed how practitioners may accidently reinforce overprotection through inadvertent use of threatening metaphors, thus, increasing pain and disability. Stilwell called for practitioners to be sensitive to how pain-related metaphors are used, reinforced, and reconceptualised when co-constructing meanings of pain for patients.

## Evidence of benefit and harm

There is a paucity of research that evaluates the efficacy of therapeutic metaphor using randomised controlled clinical trial (RCT) methodology, and we failed to find any systematic reviews of RCTs specifically evaluating therapeutic metaphor for pain. A small study by Bahremand et al. (92) found that metaphor therapy (*n* = 10) was inferior to relaxation training (*n* = 13) at alleviating pain and beliefs of hopelessness in patients with non-cardiac chest pain. Metaphor therapy was delivered in four x 2-hour sessions using two metaphoric stories designed to challenge existing beliefs, followed by discussions about the connection between the metaphoric story and the medical condition, with instructions to mentally rehearse the metaphors daily.

Gallagher et al. (93) found that delivering pain education material through metaphor and story (i.e., via a book of metaphors) assisted reconceptualization of pain and reduced catastrophizing for at least three months when delivered as a precursor to other interventions that target functional capacity. A mixed-methods systematic review of 12 RCTs (*n* = 755 participants) and four qualitative studies (*n* = 50 participants) by Watson et al. (94) demonstrated that allowing patients to tell their pain stories was a key component of success for pain science education. However, Louw et al. (95) found that overall messages of reconceptualising pain were more important than any individual story or metaphor.

A systematic review of six qualitative studies by Stewart and Ryan (96) offers indirect evidence that metaphors help people fashion meaning to pain and this assists expression of pain experience to others. Four therapeutic themes emerged for the value of metaphors for people with pain:

• Expression (relief in finding a way of expressing pain)
• Connection (repairing connections between a sense of self and culture and society)
• Understanding (to make sense of pain experience)
• Control (to express a need to regain control of life with pain).

There was insufficient evidence from the qualitative studies to judge whether the use of metaphor affected pain, function, sleep, or mood, although findings suggested that metaphors improved knowledge and understanding, communication, self-efficacy, resilience, empowerment, and behavioural change.

The possibility of adverse effects associated with the use of therapeutic metaphors has been overlooked in trials to date. Thus, evaluations of the benefits and harms of therapeutic metaphors are needed to inform their value and utility in clinical practice.

## Future directions

Concerns have been expressed that the biopsychosocial model of pain perpetuates a reductionist approach, creating artificial boundaries between biological, psychological, and social dimensions, fragmenting a person's sense of coherence, and lived experience of pain (91, 97). Carefully crafted metaphors have potential to reconstruct a person's sense of coherence. Advances in phenomenology and cognitive sciences suggest that sense-making emerges from relational processes distributed across the brain-body-environment providing opportunities to develop metaphors to capture and integrate contextual factors in sense-making of embodied and embedded aspects of pain experience in clinical and non-clinical settings.

### A role for enactive metaphors?

Enactivism is a theory for sense-making grounded in the idea that people are embodied and action-oriented beings. Enactivism is defined as *“… a relational and emergent process of sense-making through a lived body that is inseparable from the world that we shape and that shapes us.”* (98) p. 637. Enactive metaphors bring metaphors into existence through actions. Metaphors are expressed *via* movement such as play to facilitate the embodiment of the metaphor through “full-body engagement”. Thus, enactive metaphors could aid the conceptualisation, construction, and internalisation of positive meanings of pain.

#### Enactive metaphors to conceptualise pain

Stilwell et al. advocate the use of enactive metaphors to assist conceptualisation of pain through the lens of “metaphordances”—connecting enactivism to a more dynamic view of metaphor (91, 98, 99). Metaphordance encompasses possibilities available to a person for action (“landscape of affordances”) specific to a person's body and experience (“field of affordances”) and life-stage and socio-cultural practices (affordance space). Stilwell et al., argue that the landscape of affordances created by society and the healthcare system constrains the field of affordances available to a person living with pain, where agency is already restricted. Thus, activities utilising enactive metaphors have the potential to open up a person's affordances, providing opportunities to conceptualise a more encompassing understanding of pain, providing opportunities to assist people on a “healing journey”.

#### Enactive metaphors to assist health and well-being

Metaphors used in patient consultation, education and rehabilitation are usually delivered by verbal dialogue where the learner “thinks through” mappings from source to target domain, and as a consequence are static, passive and disembodied, i.e., “sitting metaphors” (24). In contrast, enactive metaphors use actions to put metaphors into existence, i.e., acting out understanding as conveyed in the metaphor. Enactive metaphors, delivered via activities such as play-acting or moving in a particular way to facilitate the embodiment of the metaphor through “full-body engagement”, reinforce learning through embodied action and help to shape how a person makes sense of their world (24). Enactive metaphors may be particularly relevant in the rehabilitation of people with persistent pain where movement and exercise are core elements of treatment, it fosters active engagement and interaction *via* embodied clinician-patient interaction.

Modern technologies using virtual and augmented realities that merge real and virtual worlds have been used to improve movement in people with fear-avoidance of pain [e.g., immersive dodgeball (100)] and to facilitate movement of artificial limbs using performance feedback [e.g., augmented reality driving of motor vehicles for phantom limb pain (101)]. Such technologies have the potential to bring enactive metaphors to life by integrating perceptions and movements to catalyse learning through body cueing (102). Gallagher and Lindgren provide evidence of the potential of enactive metaphors combined with modern technologies, including virtual reality environments to improve learning in educational settings (24). The use of motion sensing, haptic feedback, and digital imagery can augment movement activities to reinforce enactive metaphors so that the learner becomes part of the system they are trying to understand (24). Examples include expressing pain through metaphorical movement, or metaphorical sound, and conceptualising pain through enabling an inside-the-body perspective.

We have used enactive metaphors in artist-led workshops to co-create stories of living with persistent pain via creative movement, resulting in improvements in health, well-being and quality of life (103). Community-based pain services that connect people living with persistent pain to pain education and community-based activities, such as artist-lead workshops, may provide opportunities not only for the use of enactive metaphors, but also for holistic support of a person's physical, mental, social and environmental needs. An example of such a service is Rethinking Pain, Bradford and Craven, England (https://rethinkingpain.org/).

### Beyond biomedical metaphors

Constructive metaphorical language reflecting contemporary understanding of pain that extends beyond a neuro-mechanistic lens is continuously growing through pain education initiatives by public and privately owned providers. Here, we demonstrate how pain metaphors can be reconfigured to assist people in pain acceptance:

• Pain as a journey: Instead of “Fighting pain” consider “Navigating pain” or “Every day is a different path on my pain journey”.
• Pain as weather: Instead of “Pain is a thunderstorm of suffering” consider “Pain is like a cloud, sometimes dark and looming, but eventually moving on” or “Just as there are rainy days, I have painful days, and like rain eventually pain will pass with time”.
• Pain as a teacher: Instead of “Pain is like school, restricting my freedom” consider “Pain teaches me resilience” or “Every flare-up is a lesson in understanding my body”.
• Pain as waves: Instead of “Shooting pain is a tsunami of suffering” consider “Pain comes in waves, sometimes big, sometimes small. I ride them as they come” or “Like a surfer, I'm learning to ride the waves of pain”.
• Pain as a companion: Instead of “Pain engulfs my entire being” consider “Pain is a part of me, not the whole me” or “My pain is a companion on this journey, but not the driver”.
• Pain as a window-pane: Instead of “Pain has shattered my entire life” consider “Pain is like a window-pane; sometimes clear, sometimes fogged, but always providing a perspective” or “Like a cracked window-pane, pain distorts but doesn't fully block the view”.

We recommend “*The malleable magic of metaphor*” by Moseley and Butler (78) for a synopsis of the development of constructive pain metaphor.

We advocate the development of metaphors mirroring modern concepts of embodied and embedded pain that utilise notions from non-biomedical disciplines (104). This provides opportunities for new perspectives and paradigm shift. For example, informational or quantum metaphors might better resonate with the subjective nature of pain, offering a less rigid objectivity. For instance, quantum superposition, where particles exist in multiple states, can be likened to patients observing all their life's temporal moments as “now”. We have used this perspective to develop a framework called Past Adversity Influencing Now (PAIN), showcasing how some people may become “ensnared” by their temporal perception of pain (105). We believe that our PAIN framework offers practitioners a chance to use quantum metaphors to help patients reframe their pain experiences. For instance, suggesting “pain arrives from the past” might prompt patients to reconsider their past experiences in their present, possibly liberating them from their previously “fixed time-lines”—a concept in time travel theories where events are predetermined and unchangeable as an outcome.

## Summary and conclusion

In summary, we offer examples of the insidious nature of pain metaphors contributing to painogenicity in society. Metaphors link pain experience personal to oneself, to entities and events in the external world. This enables people to make sense of their own pain and to share the private world of their pain with others. Pain conversation steeped in warmongering and destructive pathoanatomical metaphor is, in some instances, detrimental to recovery. Thus, we advocate reconfiguring pain language towards constructive metaphors that encourages society to adopt a salutogenic view of pain that focuses on health and well-being.

We demonstrated that metaphors are more than figurative language; metaphors are fundamental tools for conceptual mapping, i.e., the way people think. Being cognisant of the pervasive use of metaphor provides an appreciation of their use in explanatory models of pain and assists development of accurate conceptual understanding and healthier language. Explanatory models built on neuro-mechanistic metaphor contribute to fallacies and misnomers about pain and has prejudiced research towards biomedical detail underpinning nociception, in the hope of eradicating pain by “preventing pain transmission [sic]”. This has been at the expense of research on the lived experience of pain and has constrained the exploration of non-medicalised strategies for recovery, especially for persistent pain.

In conclusion, it is a metaphorical battle—literally! Metaphors are the building blocks of conceptual understanding and have created the framework on which the science of pain is based. Metaphors spread as memes (i.e., ideas, behavior, or styles that pass from one individual to another by imitation) for acceptance in the societal narrative, constraining diverse thinking and possible alternatives. In the book The Meme Machine, Blackmore states:

“Memes spread themselves around indiscriminately without regard to whether they are useful, neutral, or positively harmful to us.” p.7 (106).

Metaphorical memes of warmongering, damage and mechanistic explanatory models gained access to the pain lexicon many centuries ago and still dominate the public understanding of the persistence of pain. Moving towards a broader eco-socio-psychological understanding of pain persistence [e.g., an ecology of wholeness view (107)] requires compelling and intuitive constructive metaphors that out-compete metaphors dominating modern-day parlance. This vocabulary-based escape route from a biomedically dominated understanding of pain offers new avenues to explore the persistence of pain within a salutogenic framework of health and well-being.

Campaigns to promote the use of positive and constructive metaphors in commercial adverts of pain interventions in corporate and social media are urgently needed. Moreover, as pain transcends all healthcare disciplines, we advocate curricula that develop the knowledge and skills needed to employ positive pain metaphors by healthcare professionals. We hope that this article catalyses debate and reflection on the sinister nature of pain metaphor, to improve conceptual understanding of pain and to purposefully promote living well with and without pain.

## References

[1] Neilson S. Pain as metaphor: metaphor and medicine. Med Humanit. (2016) 42(1):3–10. 10.1136/medhum-2015-010672 26253331 PMC4819656

[2] Lakoff G Johnson M. Metaphors we live by. London: University of Chicago Press (1980).

[3] Lakoff G. The contemporary theory of metaphor. Metaphor and thought. In: Ortony A, editors. Metaphor and thought. Cambridge: Cambridge University Press (1993). p. 202–51.

[4] Whorf BL. Language, thought, and reality: Selected writings of Benjamin Lee Whorf. Eastford, Connecticut: Martino Fine Books (2011).

[5] Sapir E. The unconscious patterning of behavior in society. In: Child CM Koffka K Anderson JE Watson JB Sapir E Thomas WI, editors. The unconscious: a symposium. New York: Alfred A. Knopf (1927). p. 114–42.

[6] Fabrega H Jr. Language, culture and the neurobiology of pain: a theoretical exploration. Behav Neurol. (1989) 2(4):235–60. 10.3233/BEN-1989-2405 24486996

[7] Boroditsky L. How language shapes thought. Sci Am. (2011) 304(2):62–5. 10.1038/scientificamerican0211-62 21319543

[8] Thibodeau PH Hendricks RK Boroditsky L. How linguistic metaphor scaffolds reasoning. Trends Cogn Sci. (2017) 21(11):852–63. 10.1016/j.tics.2017.07.001 28789831

[9] Johnson MI. Opinions on paleolithic physiology living in painogenic environments: changing the perspective through which we view chronic pain. Pain Manag. (2019) 9(3):219–24. 10.2217/pmt-2018-0095 31141471

[10] Johnson MI. The landscape of chronic pain: broader perspectives. Medicina (Kaunas). (2019) 55(5):182. 10.3390/medicina55050182 31117297 PMC6572619

[11] Borsook D Youssef AM Simons L Elman I Eccleston C. When pain gets stuck: the evolution of pain chronification and treatment resistance. Pain. (2018) 159(12):2421–36. 10.1097/j.pain.0000000000001401 30234696 PMC6240430

[12] Johnson MI Woodall J. A healthy settings approach to addressing painogenic environments: new perspectives from health promotion. Front Pain Res. (2022) 3:1000170. 10.3389/fpain.2022.1000170 PMC9551298 36238350

[13] Mittelmark M Bauer G. The meanings of salutogenesis. In: Mittelmark M Sagy S Eriksson M Bauer G Pelikan J Lindström B editors. The handbook of salutogenesis 2017. Cham, CH: Springer (2016). p. 7–13.

[14] Antonovsky A. Health stress and coping. San Francisco, US: Jossey-Bass inc (1979).

[15] Hochwälder J. Sense of coherence: notes on some challenges for future research. Sage Open. (2019) 9:1–8. 10.1177/215824401984668734290901

[16] Eriksson M Lindstrom B. A salutogenic interpretation of the Ottawa charter. Health Promot Int. (2008) 23(2):190–9. 10.1093/heapro/dan014 18356285

[17] Gaudet T. Cultural transformation to a whole health system: lessons learned. Glob Adv Health Med. (2022) 11:2164957X221091452. 10.1177/2164957221091452 35478714 PMC9036378

[18] Jonas WB Eisenberg D Hufford D Crawford C. The evolution of complementary and alternative medicine (CAM) in the USA over the last 20 years. Forsch Komplementmed. (2013) 20(1):65–72. 10.1159/000348284 23727764

[19] Oliveira CC. Suffering and salutogenesis. Health Promot Int. (2015) 30(2):222–7. 10.1093/heapro/dau061 25073763

[20] Nie JB Gilbertson A de Roubaix M Staunton C van Niekerk A Tucker JD Healing without waging war: beyond military metaphors in medicine and HIV cure research. Am J Bioeth. (2016) 16(10):3–11. 10.1080/15265161.2016.1214305 27653388 PMC5064845

[21] Lucas DW. Aristotle poetics. Oxford: Clarendon Press (1968).

[22] Lea D Bradbery L. Oxford Advanced learner’s dictionary. Oxford: Oxford University Press (2020). [20 March 2023]. Available from: https://www.oxfordlearnersdictionaries.com/definition/english/metaphor#:∼:text=%E2%80%8Ba%20word%20or%20phrase,of%20such%20words%20and%20phrases

[23] Madsen MW. Cognitive metaphor theory and the metaphysics of immediacy. Cogn Sci. (2016) 40(4):881–908. 10.1111/cogs.12320 26523770

[24] Gallagher S Lindgren R. Enactive metaphors: learning through full-body engagement. Educ Psychol Rev. (2015) 27:391–404. 10.1007/s10648-015-9327-1

[25] Lakoff G. Mapping the brain’s metaphor circuitry: metaphorical thought in everyday reason. Front Hum Neurosci. (2014) 8:958. 10.3389/fnhum.2014.00958 25566012 PMC4267278

[26] Ricoeur P. Creativity in language: word, polysemy, metaphor. Philosophy Today. (1973) 17:1.

[27] Clarke A Anthony G Gray D Jones D McNamee P Schofield P I feel so stupid because I can't give a proper answer…” how older adults describe chronic pain: a qualitative study. BMC Geriatr. (2012) 12:78. 10.1186/1471-2318-12-78 23276327 PMC3544685

[28] Nortvedt F Engelsrud G. “Imprisoned” in pain: analyzing personal experiences of phantom pain. Med Health Care Philos. (2014) 17(4):599–608. 10.1007/s11019-014-9555-z 24647898 PMC4182588

[29] Biro D. The language of pain: finding words, compassion and relief. New York: W.W. Norton & Company Ltd (2010).

[30] Bourke J. Pain: metaphor, body, and culture in anglo-American societies between the eighteenth and twentieth centuries. Rethink Hist. (2014) 18(4):475–98. 10.1080/13642529.2014.893660 28331427 PMC5324398

[31] Cohen M Weisman A Quintner J. Pain is not a “thing": how that error affects language and logic in pain medicine. J Pain. (2022) 23(8):1283–93. 10.1016/j.jpain.2022.03.235 35427806

[32] Cohen M Weisman A Quintner J. Response to van Rysewyk S and Moseley GL et al.’s comments on Cohen et al. J Pain 2022; 23(8):1283-1293. J Pain. (2023) 24(1):184–5. 10.1016/j.jpain.2022.11.003 36400173

[33] Weisman A Quintner J Galbraith M Masharawi Y. Why are assumptions passed off as established knowledge? Med Hypotheses. (2020) 140:109693. 10.1016/j.mehy.2020.109693 32234641

[34] Bourke J. The story of pain. From prayers to painkillers. New York, USA: Oxford University Press (2014).

[35] Semino E. Descriptions of pain, metaphor and embodied simulation. Metaphor Symb. (2010) 25(4):205–26. 10.1080/10926488.2010.510926

[36] Shinebourne P Smith JA. The communicative power of metaphors: an analysis and interpretation of metaphors in accounts of the experience of addiction. Psychol Psychother. (2010) 83(1):59–73. 10.1348/147608309X468077 19712543

[37] McFarland L Barlow J Turner A. Understanding metaphor to facilitate emotional expression during a chronic disease self-management course. Patient Educ Couns. (2009) 77(2):255–9. 10.1016/j.pec.2009.03.024 19395228

[38] Munday I Newton-John T Kneebone I. Clinician experience of metaphor in chronic pain communication. Scand J Pain. (2022). 23(1):88–96. PMID: 35920187. 10.1515/sjpain-2022-0043 35920187

[39] Díaz Vera JE. When pain is not a place: pain and its metaphors in late middle English medical texts. Onomázein. (2012) 26(2):279–308. 10.7764/onomazein.26.10 (Accessed September 8, 2023).

[40] Cohen E. The modulated scream: pain in late medieval culture. London: University of Chicago Press Ltd (2010). 384.

[41] Bourke J. Pain, sympathy and the medical encounter between the mid eighteenth and the mid twentieth centuries. Hist Res. (2012) 85(229):430–52. 10.1111/j.1468-2281.2011.00593.x 24489439 PMC3906540

[42] Munday I Newton-John T Kneebone I. ‘Barbed wire wrapped around my feet': metaphor use in chronic pain. Br J Health Psychol. (2020) 25(3):814–30. 10.1111/bjhp.12432 32452109 PMC7496857

[43] Raja SN Carr DB Cohen M Finnerup NB Flor H Gibson S The revised international association for the study of pain definition of pain: concepts, challenges, and compromises. Pain. (2020) 161(9):1976–82. 10.1097/j.pain.0000000000001939 32694387 PMC7680716

[44] Beecher HK. Anesthesia for men wounded in battle. Ann Surg. (1945) 122(5):807–19. 10.1097/00000658-194511000-00004 17858686 PMC1618301

[45] Fisher J Hassan D O’ Connor N. Minerva. Br Med J. (1995) 310:70. 10.1136/bmj.310.6971.70

[46] Bayer TL Baer PE Early C. Situational and psychophysiological factors in psychologically induced pain. Pain. (1991) 44(1):45–50. 10.1016/0304-3959(91)90145-N 2038488

[47] Castro WH Meyer SJ Becke ME Nentwig CG Hein MF Ercan BI No stress–no whiplash? Prevalence of “whiplash” symptoms following exposure to a placebo rear-End collision. Int J Legal Med. (2001) 114(6):316–22. 10.1007/s004140000193 11508796

[48] Lane HP McLachlan S Philip J. The war against dementia: are we battle weary yet? Age Ageing. (2013) 42(3):281–3. 10.1093/ageing/aft011 23446313

[49] Wiggins NM. Stop using military metaphors for disease. Br Med J. (2012) 345:e4706. 10.1136/bmj.e4706 22791794

[50] McCartney M. The fight is on: military metaphors for cancer may harm patients. Br Med J. (2014) 349:g5155. 10.1136/bmj.g5155 25128488

[51] Eccleston C Crombez G. Worry and chronic pain: a misdirected problem solving model. Pain. (2007) 132(3):233–6. 10.1016/j.pain.2007.09.014 17961924

[52] Corkhill B. Pain signals and other bad language. In: Wemyss-Gorman P, editors. Innovative approaches to chronic pain. London and Philadelphia: Jessica Kingsley Publishers (2021). p. 119–32.

[53] Scarry E. The body in pain: the making and unmaking of the world. USA: Oxford University Press (1985).

[54] Barbiero G Berto R. Biophilia as evolutionary adaptation: an onto- and phylogenetic framework for biophilic design. Front Psychol. (2021) 12:700709. 10.3389/fpsyg.2021.700709 34367025 PMC8334556

[55] Halliburton M. “Just some spirits": the erosion of spirit possession and the rise of “tension” in south India. Med Anthropol. (2005) 24(2):111–44. 10.1080/01459740590933849 16019568

[56] Halliburton M. Hegemony versus pluralism: Ayurveda and the movement for global mental health. Anthropol Med. (2023) 30(2):85–102. 10.1080/13648470.2020.1785842 32873052

[57] Halliburton M. Finding a fit: psychiatric pluralism in south India and its implications for who studies of mental disorder. Transcult Psychiatry. (2004) 41(1):80–98. 10.1177/1363461504041355 15171208

[58] Halliburton M. Rethinking anthropological studies of the body: manas and bōdham in kerala. Am Antropol. (2002) 104(4):1123–34. 10.1525/aa.2002.104.4.1123

[59] Fromm E. The anatomy of human destructiveness. London: Pimlico (1997).

[60] Lenton TM Dutreuil S Latour B. Life on earth is hard to spot. Anthr Rev. (2020) 7:248–72. 10.1177/2053019620918939

[61] Shneidman ES. Suicide as psychache. J Nerv Ment Dis. (1993) 181(3):145–7. 10.1097/00005053-199303000-00001 8445372

[62] Sheldrake R. The science delusion. London: Hodder & Stoughton Ltd (2020).

[63] Wall PD. The gate control theory of pain mechanisms. A re-examination and re-statement. Brain. (1978) 101(1):1–18. 10.1093/brain/101.1.1 205314

[64] Melzack R Wall PD. Pain mechanisms: a new theory. Science. (1965) 150(3699):971–9. 10.1126/science.150.3699.971 5320816

[65] Johnson MI Bonacaro A Georgiadis E Woodall J. Reconfiguring the biomedical dominance of pain: time for alternative perspectives from health promotion? Health Promot Int. (2022) 37(4):1–9. 10.1093/heapro/daac128 36102475

[66] Sik D. Power from indirect pain: a historical phenomenology of medical pain management. Cont Philos Rev. (2021) 54:41–59. 10.1007/s11007-020-09518-5

[67] Bendelow G. Chronic pain patients and the biomedical model of pain. Virtual Mentor. (2013) 15(5):455–9. 10.1001/virtualmentor.2013.15.5.msoc1-1305 23680569

[68] Corns J. The complex reality of pain. London: Routledge (2022).

[69] Coninx S. The notorious neurophilosophy of pain: a family resemblance approach to idiosyncrasy and generalizability. Mind Lang. (2023) 38(1):178–97. 10.1111/mila.12378

[70] Zhang W Lyu M Bessman NJ Xie Z Arifuzzaman M Yano H Gut-innervating nociceptors regulate the intestinal microbiota to promote tissue protection. Cell. (2022) 185(22):4170–89 e20. 10.1016/j.cell.2022.09.008 36240781 PMC9617796

[71] Panteleev SS Sivachenko IB Lyubashina OA. The buspirone-dependent abdominal pain transmission within the nucleus tractus solitarius in the rat. Neuroscience. (2021) 452:326–34. 10.1016/j.neuroscience.2020.11.032 33248152

[72] Xu Q Ford NC He S Huang Q Anderson M Chen Z Astrocytes contribute to pain gating in the spinal cord. Sci Adv. (2021) 7(45):eabi6287. 10.1126/sciadv.abi6287 34730998 PMC8565904

[73] Nature. The surprise saviours of the gut: the neurons that sense pain. Nature. (2022) 611:12. 10.1038/d41586-022-03320-4

[74] Violet TK. Constructing the gendered risk of illness in lyrica ads for fibromyalgia: fear of isolation as a motivating narrative for consumer demand. J Med Humanit. (2022) 43(1):55–64. PMID: 31475311. 10.1007/s10912-019-09575-9 31475311

[75] Clow B. Who’s afraid of Susan Sontag? Or, the myths and metaphors of cancer reconsidered. Soc Hist Med. (2001) 14(2):293–312. 10.1093/shm/14.2.293 11697354

[76] Sontag S. Illness as metaphor. New York City, NY: Farrar, Straus and Giroux (1978).

[77] Moseley GL Pearson N Reezigt R Madden VJ Hutchinson MR Dunbar M Considering precision and utility when we talk about pain. Comment on Cohen et al. J Pain. (2023) 24(1):178–81. 10.1016/j.jpain.2022.05.010 36549800

[78] Moseley GL Butler DS. The malleable magic of metaphor. In: Moseley GL Butler DS, editors. Explain pain supercharged the clinician’s manual. Adelaide, Australia: Noigroup Publications (2017). p. 143–67.

[79] Butler DS Moseley GL. Explain pain. Adelaide: Noigroup Publications (2013).

[80] Williams AC. What can evolutionary theory tell US about chronic pain? Pain. (2016) 157(4):788–90. 10.1097/j.pain.0000000000000464 26683235

[81] Price TJ Dussor G. Evolution: the advantage of ‘maladaptive’ pain plasticity. Curr Biol. (2014) 24(10):R384–6. 10.1016/j.cub.2014.04.011 24845663 PMC4295114

[82] Walters ET Williams ACC. Evolution of mechanisms and behaviour important for pain. Philos Trans R Soc Lond B Biol Sci. (2019) 374(1785):20190275. 10.1098/rstb.2019.0275 31544614 PMC6790384

[83] Moseley GL. Painful yarns: metaphors and stories to help understand the biology of pain. Canberra: Dancing Giraffe Press (2010).

[84] Storr W. The science of stroytelling. London: Williams Collins (2019).

[85] Ramirez ES Ruiz FJ Pena-Vargas A Bernal PA. Empirical investigation of the verbal cues involved in delivering experiential metaphors. Int J Environ Res Public Health. (2021) 18(20):10630. PMID: 34682375; PMCID: PMC8535567. 10.3390/ijerph182010630 34682375 PMC8535567

[86] Stones C Cole F. Breaking the cycle: extending the persistent pain cycle diagram using an affective pictorial metaphor. Health Commun. (2014) 29(1):32–40. 10.1080/10410236.2012.715537 23356651

[87] Vilardaga R Davies PS Vowles KE Sullivan MD. Theoretical grounds of pain tracker self manager: an acceptance and commitment therapy digital intervention for patients with chronic pain. J Contextual Behav Sci. (2020) 15:172–80. 10.1016/j.jcbs.2020.01.001 32269915 PMC7141572

[88] Padfield D Zakrzewska JM Williams AC. Do photographic images of pain improve communication during pain consultations? Pain Res Manag. (2015) 20(3):123–8. 10.1155/2015/145964 25996763 PMC4447153

[89] Padfield D Omand H Semino E Williams ACC Zakrzewska JM. Images as catalysts for meaning-making in medical pain encounters: a multidisciplinary analysis. Med Humanit. (2018) 44(2):74–81. 10.1136/medhum-2017-011415 29895594 PMC6031279

[90] Padfield D Janmohamed F Zakrzewska JM Pither C Hurwitz B. A slippery surface…can photographic images of pain improve communication in pain consultations? Int J Surg. (2010) 8(2):144–50. 10.1016/j.ijsu.2009.11.014 20064481

[91] Stilwell P Stilwell C Sabo B Harman K. Painful metaphors: enactivism and art in qualitative research. Med Humanit. (2021) 47:235–47. 10.1136/medhum-2020-011874 33077504

[92] Bahremand M Moradi G Saeidi M Mohammadi S Komasi S. Reducing irrational beliefs and pain severity in patients suffering from non-cardiac chest pain (Nccp): a comparison of relaxation training and metaphor therapy. Korean J Pain. (2015) 28(2):88–95. 10.3344/kjp.2015.28.2.88 25852829 PMC4387467

[93] Gallagher L McAuley J Moseley GL. A randomized-controlled trial of using a book of metaphors to reconceptualize pain and decrease catastrophizing in people with chronic pain. Clin J Pain. (2013) 29(1):20–5. 10.1097/AJP.0b013e3182465cf7 22688603

[94] Watson JA Ryan CG Cooper L Ellington D Whittle R Lavender M Pain neuroscience education for adults with chronic musculoskeletal pain: a mixed-methods systematic review and meta-analysis. J Pain. (2019) 20(10):1140 e1–e22. 10.1016/j.jpain.2019.02.011 30831273

[95] Louw A Puentedura EJ Diener I Zimney KJ Cox T. Pain neuroscience education: which pain neuroscience education metaphor worked best? S Afr J Physiother. (2019) 75(1):1329. 10.4102/sajp.v75i1.1329 31535053 PMC6739553

[96] Stewart M Ryan S-J. Do metaphors have therapeutic value for people in pain? a systematic review pain and rehabilitation. J Physio Pain Assoc. (2019) 2020(48):10–23.

[97] Sullivan MD Sturgeon JA Lumley MA Ballantyne JC. Reconsidering fordyce’s classic article, “Pain and suffering: what is the unit?” to help make our model of chronic pain truly biopsychosocial. Pain. (2023) 164(2):271–9. 10.1097/j.pain.0000000000002748 35972469 PMC9840653

[98] Stilwell P Harman K. An enactive approach to pain: beyond the biopsychosocial model. Phenomenol Cogn Sci. (2019) 18:637–65. 10.1007/s11097-019-09624-7

[99] Cormack B Stilwell P Coninx S Gibson J. The biopsychosocial model is lost in translation: from misrepresentation to an enactive modernization. Physiother Theory Pract. (2022) 28:1–16. PMID: 35645164. 10.1080/09593985.2022.2080130 [Epub ahead of print]. 35645164

[100] Thomas JS France CR Applegate ME Leitkam ST Walkowski S. Feasibility and safety of a virtual reality dodgeball intervention for chronic low back pain: a randomized clinical trial. J Pain. (2016) 17(12):1302–17. 10.1016/j.jpain.2016.08.011 27616607 PMC5125833

[101] Ortiz-Catalan M Sander N Kristoffersen MB Hakansson B Branemark R. Treatment of phantom limb pain (PLP) based on augmented reality and gaming controlled by myoelectric pattern recognition: a case study of a chronic PLP patient. Front Neurosci. (2014) 8:24. 10.3389/fnins.2014.00024 24616655 PMC3935120

[102] Lindgren R Tscholl M Wang S Johnson E. Enhancing learning and engagement through embodied interaction within a mixed reality simulation. Comput Educ. (2016) 95:174–87. 10.1016/j.compedu.2016.01.001

[103] Johnson MI Chazot P Cole F Cruickshank R Fuller D Keyse C Pain through the perspective of art and creativity: insights from the unmasking pain project. Front Pain Res (Lausanne). (2023) 4:1179116. 10.3389/fpain.2023.1179116 37325675 PMC10267741

[104] Tabor A Keogh E Eccleston C. Embodied pain-negotiating the boundaries of possible action. Pain. (2017) 158(6):1007–11. 10.1097/j.pain.0000000000000875 28195855

[105] Hudson M Johnson MI. Past Adversity Influencing Now (PAIN): perspectives on the impact of temporal language on the persistence of pain. Front Pain Res. (2023). 10.3389/fpain.2023.1217721 PMC10544332 37790120

[106] Blackmore S. The meme machine. Oxford: Oxford University Press (1999).

[107] Agarwal V. Medical humanism, chronic illness, and the body in pain: an ecology of wholeness. Lanham, MD: Lexington Books (2020). 328.

